# Human Leukocyte Antigen Class II associations in late‐onset Myasthenia Gravis

**DOI:** 10.1002/acn3.51309

**Published:** 2021-02-05

**Authors:** Gregorio Spagni, Laura Todi, Gabriele Monte, Mariagrazia Valentini, Gabriele Di Sante, Valentina Damato, Mariapaola Marino, Amelia Evoli, Francesca Lantieri, Carlo Provenzano

**Affiliations:** ^1^ Dipartimento di Neuroscienze Sezione di Neurologia Università Cattolica del Sacro Cuore Fondazione Policlinico Universitario “A. Gemelli” IRCCS Rome Italy; ^2^ Dipartimento di Medicina e chirurgia traslazionale Università Cattolica del Sacro Cuore Fondazione Policlinico Universitario “A. Gemelli” IRCCS Sezione di Patologia generale Rome Italy; ^3^ U.O.C. di Neurologia Fondazione Policlinico Universitario “A. Gemelli” IRCCS Roma Italy; ^4^ Dipartimento di Scienze della Salute Università degli Studi di Genova Genova Italy

## Abstract

**Objective:**

Genetic factors predisposing to late‐onset myasthenia gravis (LOMG) have not been clearly defined yet. However, genome‐wide association studies identified Human Leukocyte Antigen (HLA) Class II alleles as a hotspot in this disease subtype. The aim of this study was to analyze the correlations of HLA Class II alleles with clinical data and titin antibodies in this patient subgroup.

**Methods:**

This study consecutively enrolled anti‐acetylcholine receptor antibody‐positive, non‐thymoma patients with generalized LOMG. All patients were of Italian ancestry. HLA‐DRB1 and ‐DQB1 genotyping and serum titin antibody testing were performed in this population.

**Results:**

A total of 107 patients (females: 28/107, 26.2%; median age of onset: 68 years, range: 50‐92) were included. We found a positive association with HLA‐DRB1*07 (*P* = 1.1 × 10^‐5^), HLA‐DRB1*14 (*P* = 0.0251) and HLA‐DQB1*02 (*P* = 0.0095). HLA‐DRB1*03, HLA‐DRB1*11, and HLA‐DQB1*03 were protective alleles (*P* = 7.9 × 10^‐5^, *P* = 0.0104, and *P* = 0.0067, respectively). By conditional haplotype analysis, HLA‐DRB1*07‐DQB1*02 was found to be the major risk haplotype (OR = 4.10; 95% C.I.: 2.80‐5.99; *P* = 6.01 × 10^‐11^). The mean age at onset was 73.4 years in DRB1*07 homozygotes, 69.7 years in heterozygotes, and 66.6 in non‐carriers (*P* = 0.0488). DRB1*07 carriers and non‐carriers did not differ in disease severity and response to therapy. Titin antibodies were detected in 61.4% of the cases, having no association with HLA alleles or specific clinical characteristics.

**Interpretation:**

In our study, we identified the HLA DRB1*07‐DQB1*02 haplotype as a predisposing factor for the development of generalized LOMG in the Italian population.

## Introduction

Myasthenia gravis (MG) is caused by antibodies (Abs) to post‐synaptic proteins at the motor end‐plate.^1^ Abs to the nicotinic acetylcholine receptor (AChR) are the most common, being detected in around 85% of MG cases.^1^ AChR MG includes three clinical subtypes: early‐onset MG (EOMG), late‐onset MG (LOMG), generally defined by a cut‐off age of 50 years, and thymoma‐associated MG.^2^ While EOMG epidemiology has not changed over time, the incidence and prevalence rates of LOMG have steadily increased in the last decades.^3^, ^4^, ^5^, ^6^ Given the aging of the general population, it is predictable that LOMG will be even more prevalent in the near future.

It is well‐known that LOMG is associated, in about 50% of the cases, with Abs to titin and, to a lesser extent, to the ryanodine receptor (RyR), which are otherwise markers of thymoma.^7^ In earlier studies, titin and RyR Abs were found to be predictive of severe MG with bulbar involvement.^8^, ^9^

MG subtypes have different Human Leukocyte Antigen (HLA) associations. In Caucasians, EOMG is strongly linked to the extended haplotype 8.1 (A1‐B8‐DQ2‐DR3) across populations,^10^, ^11^, ^12^, ^13^ whereas LOMG has been found to be associated with several different Class II alleles.^12^, ^14^, ^15^, ^16^, ^17^, ^18^, ^19^ Genome‐wide association studies (GWAS) confirmed the relevance of HLA Class II region, although no single high‐risk variant was identified.^20^, ^21^ Inherent heterogeneity of LOMG, differences in studies on inclusion criteria, age cut‐off, patient stratification and sample size, and, not least, the strong linkage disequilibrium among HLA alleles, most likely add to the complexity of this research area.

The aim of this study was to investigate Class II HLA alleles, and their correlation with clinical data and titin Abs, in an Italian cohort of AChR‐positive LOMG patients.

## Methods

### Patient population

Patients were enrolled in the study during follow‐up visits from March 2019 to March 2020 according to the following criteria: (1) generalized AChR‐positive MG; (2) absence of thymoma; (3) disease onset ≥50 years of age; (4) Italian ancestry; (5) disease duration ≥2 year.

The clinical diagnosis of MG was confirmed in all patients by positive results of anti‐AChR Abs tested by radioimmunoassay (RSR Ltd, Cardiff, UK). Patients’ information was collected retrospectively from chart review and prospectively during the study. MG treatment was performed according to accepted guidelines and common clinical practice.^22^ Disease severity and response to treatment were graded with the MG Foundation of America (MGFA) system.^23^ All patients underwent contrast‐enhanced chest CT scan to rule out the presence of thymoma. In six patients who underwent thymectomy, histological examination showed no evidence of thymoma.

To verify that the included cohort was representative of our total LOMG population, we performed a preliminary comparative analysis of the main clinical and demographic features of the included cohort versus the total LOMG population treated at our Center (n = 254) (Table S1).

### HLA genotyping and titin antibody testing

Whole blood samples were collected in EDTA tubes and stored at −20°C until further use. Genomic DNA was extracted using QIAamp DNA Blood Mini Kit (Qiagen, Hilden, Germany) according to manufacturer's instruction and eluted in 100 µL of Tris‐EDTA buffer, then stored in aliquots at −20°C.

HLA typing was performed using a reverse line blot method (INNO‐LiPA HLA‐DRB1 Plus, INNO‐LiPA HLA‐DQB1 UPDATE assay kits respectively; Innogenetics, Dartford, UK), according to the manufacturer’s instructions.

Patients’ serum samples were stored at −20°C until further use. Sera were tested for titin Abs by a commercially available ELISA kit (DLD Diagnostika, Hamburg, Germany), according to manufacturer’s instructions; positivity was assessed as per instructions. This test gives only a positive/negative result.

### Statistical analysis

Summary statistics were reported as mean (± standard deviation, SD) or median (with range) for continuous variables and as numbers and percentages for categorical variables. For comparisons between the groups, the Chi‐square test, the Fisher’s Exact Test, and the Mann–Whitney U test were performed, as appropriate. Allelic and haplotype associations were analyzed individually versus all the others pooled together, applying the Fisher’s exact test and correcting the p‐value for multiple testing with the False Discovery Rate (FDR), Benjamini–Hochberg method with R.^24^ As control population for the allelic associations, we used the one reported by Rendine et al. for random Italian normal subjects genotyped for the DRB1 locus (n = 22114) and for the DQB1 locus (n = 2087),^25^ which corresponds to Italypop5 in Allele Frequency Net Database (http://www.allelefrequencies.net). For the haplotype comparisons, we estimated the haplotype counts for the DRB1 and DQB1 loci from the Allele Frequency Net Database, searching for the haplotype frequency with any DRB1 and any DQB1 allele of the Italypop5 population^25^ available in the database (n = 1447 patients genotyped for DRB1 and DQB1, with a missingness of 2.5%). Reconstruction and estimates of haplotype frequencies for MG patients were performed using PHASE 2.1.^26^ To check whether such an unbalanced ratio in the sample size of patients and controls could affect statistical analysis, we simulated 100 control samples with the same size as in cases (214 alleles) for the main association results (i.e. DRB1*07, DQB1*02 and the DRB1*07‐DQB1*02 haplotype). Finally, we carried out a conditional analysis focusing on the DRB1*07 and DQB1*02 alleles, and assessed the Linkage Disequilibrium (LD) between the two loci with Plink (http://pngu.mgh.harvard.edu/purcell/plink/).^27^ Furthermore, we performed an association analysis between DRB1*07 and age at onset and between DRB1*07 and titin positivity.

### Standard protocol approvals, registrations, and patient consents

All patients provided written informed consent to the study, conducted according to the Helsinki declaration and approved by the Ethic Committee of the Università Cattolica del Sacro Cuore (Rome, Italy) with E.C. protocol number 49886/18 (9024/19, ID:2327).

## Results

**Figure 1:**
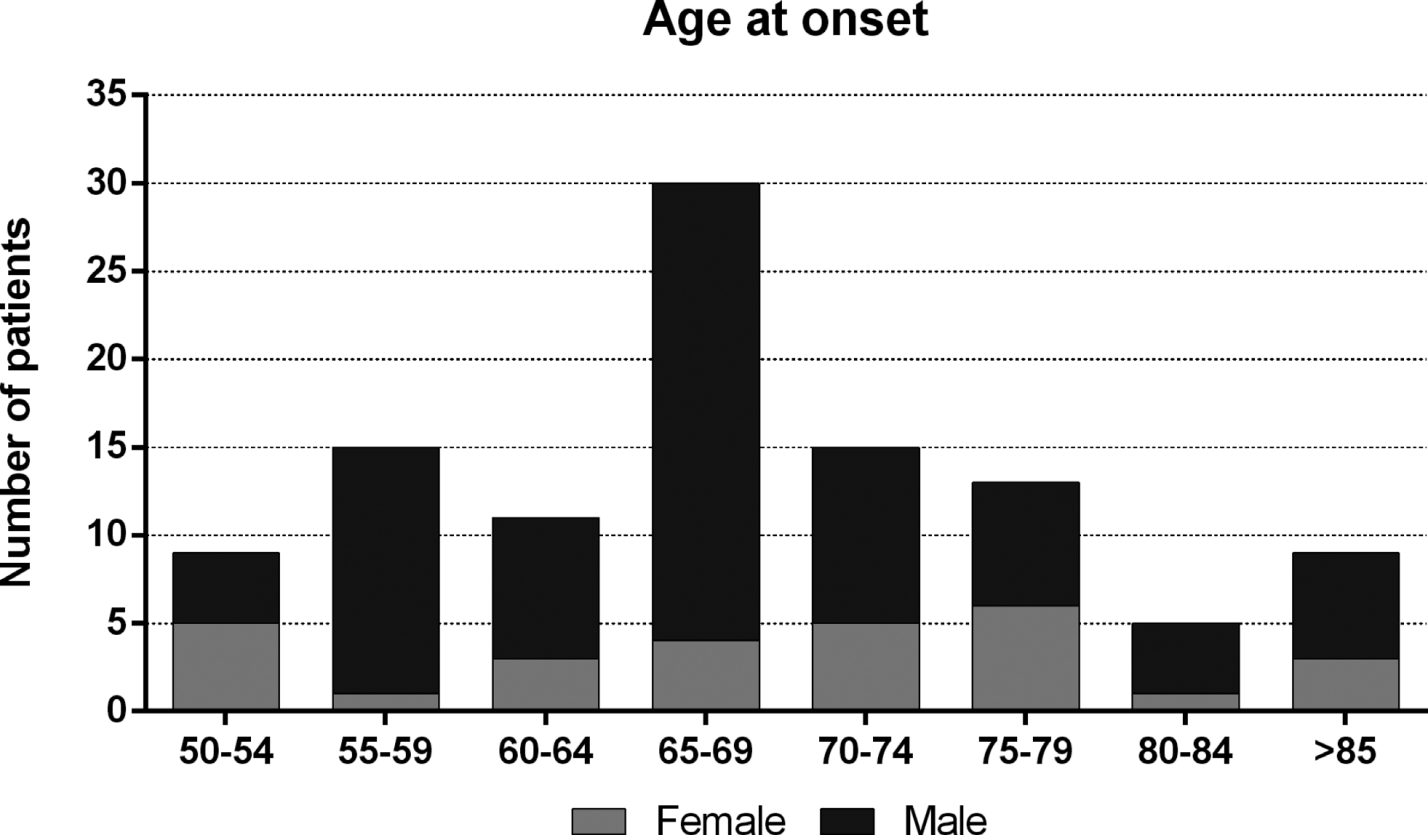
Age at onset of LOMG patients. The figure shows the number of female and male patients according to age at disease onset.

This study included 107 patients, with age at onset ranging 50 to 92 years (median: 68 years) and a clinical follow‐up ranging 2 to 24 years (median: 5 years). Demographic and clinical features are summarized in Table 1. We observed a clear male predominance (female to male ratio = 1:2.8), with females outnumbering males only among patients with onset between 50 and 54 years (Fig. 1). All patients were of Italian ancestry, mostly from Central and Southern Italy (Table S1). The comparative analysis showed that the cohort included in the study was representative of the total LOMG population (n = 254) treated at our Center (Table S1).

**Table 1:** Demographic and clinical characteristics of LOMG patients included in the study.

Included LOMG Cohort N = 107	
Female	28 (26.2%)
Median age at onset (range), years	68 (50‐92)
Median follow‐up (range), years	5 (2‐24)
Titin Ab positive	43 (61.4%)*
Max. MGFA	
II	38 (35.5%)
III	39 (36.5%)
IV	15 (14%)
V	15 (14%)
Relevant comorbidities	
Other autoimmune diseases	13 (12.1%)
Tumors	18 (16.8%)
Neurodegenerative diseases	4 (3.7%)
Therapy used	
Pyridostigmine	11 (10.3%)
Steroids	22 (20.6%)
Steroids + IS	74 (69.2%)
Post‐intervention Status	
CSR	4 (3.7%)
MM/PR	72 (67.3%)
I	28 (26.2%)
U, W, D	3 (2.8%)

LOMG, late‐onset myasthenia gravis; Ab, antibody; MGFA, Myasthenia Gravis Foundation of America classification; IS, immunosuppressor; CSR, complete stable remission; MM, minimal‐manifestations; PR, pharmacologic remission; D, Died of MG; W, worsened; U, unchanged; I, improved.

* Titin Abs were tested only in 70 patients.

Table 1 shows maximum disease severity and treatment. Most patients had mild to moderate disease (MGFA class II‐III in 77/107 cases, 71.9%) and 96/107 (89.7%) required immunosuppressive therapy based on steroids and other immunosuppressants, at least at some time during the course of their disease. Six patients (5.6%) underwent thymectomy and the histopathological analysis showed a normal‐for‐age atrophic thymus in four of them and mild thymic hyperplasia in the other two. At the end of the observation period, 76/107 (71%) patients had achieved a status of “minimal manifestations” or better. Complete stable remission was rare, accounting for 4/107 (3.7%) cases.

**Figure 2:**
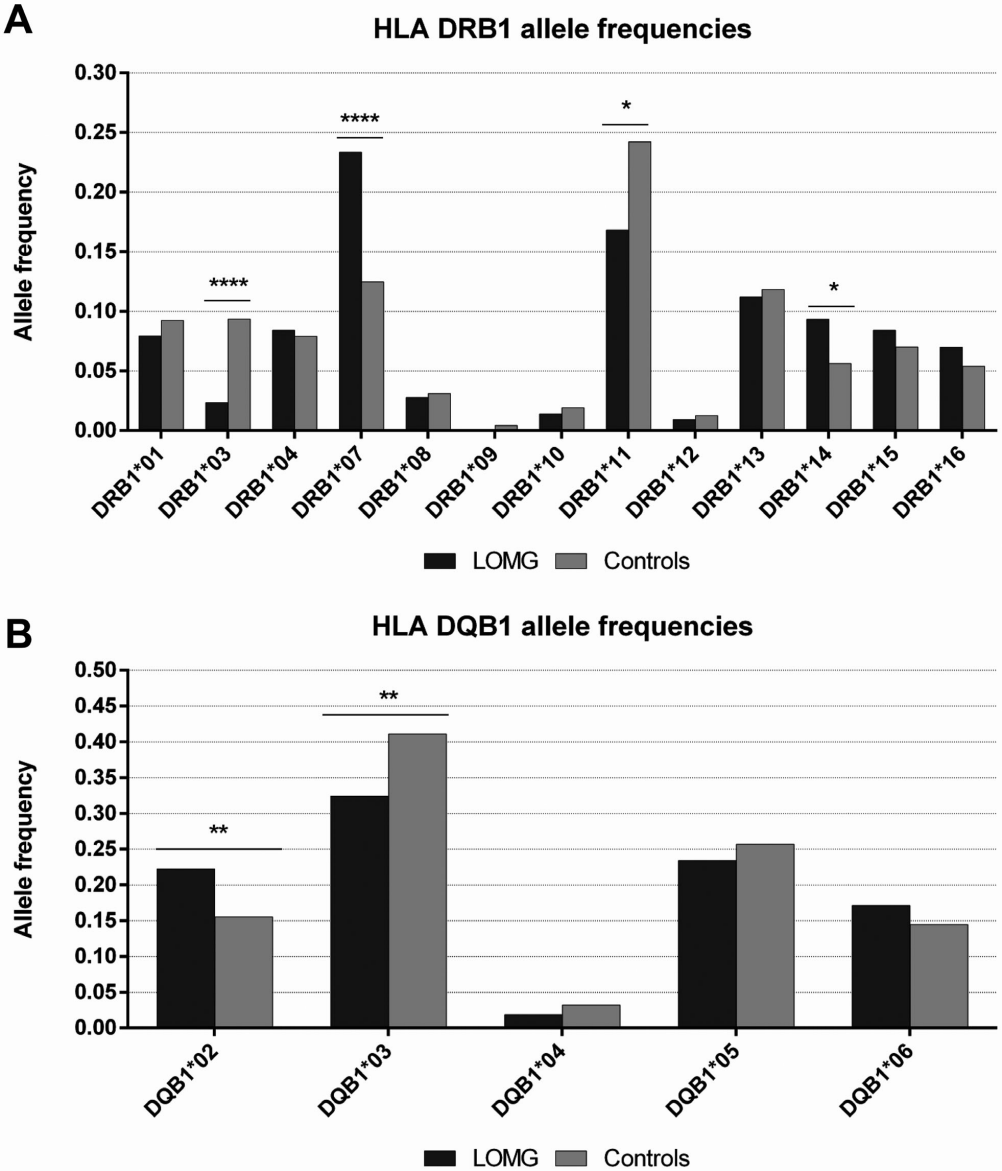
HLA Class II allele frequencies. The figure shows HLA DRB1 (A) and DQB1 (B) allele frequencies among LOMG patients and controls. Nominal p‐values are indicated by asterisks: *P < 0.05, **P < 0.01, and ****P < 0.0001.

HLA‐DRB1 allele frequencies of patients and controls are reported in Figure 2A and in Table 2. We found a significant positive association with HLA‐DRB1*07 (OR = 2.14; 95% C.I. 1.55‐2.94; p = 1.08x10^‐5^), and a weaker one with DRB1*14 (OR = 1.73; 95% C.I. 1.09‐2.74; p = 0.0251). We found a negative association with HLA‐DRB1*11 (OR = 0.63; 95% C.I.: 0.44‐0.91; p = 0.0104) and with HLA‐DRB1*03 (OR = 0.23; 95% C.I. 0.10‐0.56; p = 7.89x10^‐5^), which had a very low frequency in our cohort (0.0234 vs 0.0936 in controls). The frequency of HLA‐DRB1*15 and HLA‐DRB1*16 alleles, which were associated with LOMG in other series,^12^, ^18^ did not differ significantly between patients and controls. The associations with DRB1*03 and DRB1*07 were still highly significant after FDR correction was carried out on the 19 alleles detected at the two loci.

**Table 2:** HLA DRB1 and DQB1 allele frequencies and absolute numbers among patients and controls.

Allele	Case frequency (#)	Control frequency (#)	OR (95% C.I.)	*P*
DRB1*01	0.0794 (17)	0.0925 (4091)		NS
DRB1*03	0.0234 (5)	0.0936 (4140)	0.23 (0.10‐0.56)	7.89 × 10^‐5^
DRB1*04	0.0841 (18)	0.0791 (3498)		NS
DRB1*07	0.2336 (50)	0.1249 (5524)	2.14 (1.55‐2.94)	1.08 × 10^‐5^
DRB1*08	0.028 (6)	0.0311 (1375)		NS
DRB1*09	0 (0)	0.0044 (195)		NS
DRB1*10	0.014 (3)	0.0193 (854)		NS
DRB1*11	0.1682 (36)	0.2422 (10712)	0.63 (0.44‐0.91)	0.0104
DRB1*12	0.0093 (2)	0.0126 (557)		NS
DRB1*13	0.1121 (24)	0.1185 (5241)		NS
DRB1*14	0.0935 (20)	0.0563 (2490)	1.73 (1.09‐2.74)	0.0251
DRB1*15	0.0841 (18)	0.0702 (3105)		NS
DRB1*16	0.0701 (15)	0.054 (2388)		NS
DQB1*02	0.2243 (48)	0.1555 (649)	1.57 (1.13‐2.19)	0.0095
DQB1*03	0.3178 (68)	0.4111 (1716)	0.67 (0.50‐0.90)	0.0067
DQB1*04	0.0187 (4)	0.0321 (134)		NS
DQB1*05	0.2664 (57)	0.2569 (1072)		NS
DQB1*06	0.1729 (37)	0.1445 (603)		NS

OR, Odds ratio; C.I., confidence interval; NS, not significant.

HLA‐DQB1 allele frequencies in patients and controls are reported in Figure 2B and in Table 2. We found a positive association with HLA‐DQB1*02 (OR = 1.57, 95% CI: 1.13‐2.19, *P* = 0.0095) and a negative one with HLA‐DQB1*03 (OR = 0.67, 95% CI: 0.50‐0.90, *P* = 0.0067). The allele frequency of HLA‐DQB1*04, *05, and *06 did not differ between patients and controls.

**Figure 3:**
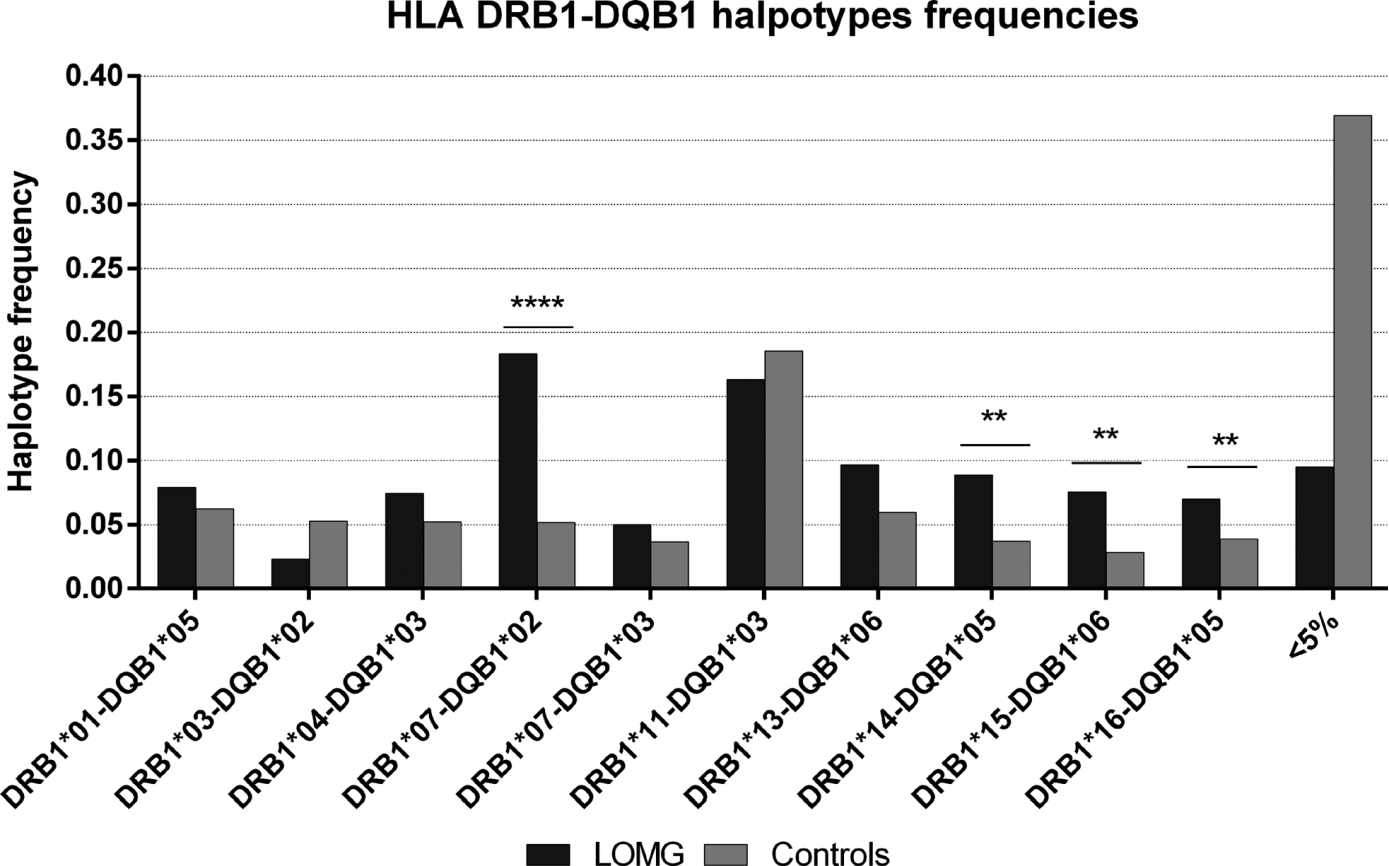
Haplotype distribution between LOMG patients and controls. Haplotypes with frequencies <5% in both groups have been pooled together. These rare haplotypes are overall much less represented among patients, most likely due to the smaller sample size and to a numerical counting effect. Nominal p‐values are indicated by asterisks: *P < 0.05, **P < 0.01, and ****P < 0.0001.

As shown in Figure 3 and Table S4, haplotype analysis found that HLA‐DRB1*07‐DQB1*02 was significantly associated with LOMG and conferred the strongest risk in this MG subtype (HLA‐DRB1*07‐DQB1*02 vs all the other haplotypes: OR = 4.10, 95% C.I. 2.80‐5.99, *P* = 6.01 × 10^‐11^). In addition, we found a positive, although weaker, association with DRB1*14‐DQB1*05 (OR = 2.45, 95% C.I. 1.47‐4.07, *P* = 0.0012) and DRB1*15‐DQB1*06 (OR = 2.70, 95% C.I. 1.55‐4.70, *P* = 0.0018) haplotypes. These associations were still significant after correction for multiple tests. To verify that the high ratio imbalance between cases and controls could not introduce any bias, and confirm the robustness of our findings, we run 100 simulations on 214 randomly selected alleles with N = 214 (sample size equal to cases) from the control dataset. We found that all the 100 simulated control sets presented allelic frequencies lower than those detected in the LOMG set (range 0.028‐0.093, being 0.052 in the whole control cohort and 0.187 in LOMG) and significant association p‐values (range 7.05 × 10^‐3^ ‐ 7.76 × 10^‐8^ vs 6.01 × 10^‐11^ obtained on the whole controls dataset). Using the same approach, we could confirm the association results for DRB1*07 (frequencies in simulated controls between 0.075 and 0.182, 0.125 in the whole control cohort and 0.234 in LOMG; p‐values from 6.83x10^‐6^ to 0.2335, 93 out of 100 < 0.05) and, to a lesser extent, for DQB1*02 (frequencies in simulated controls between 0.107 and 0.220, 0.125 in the whole control cohort and 0.224 in LOMG; p‐values from 0.0017 to 1.00, 42 out of 100 *P*‐values < 0.05).

Since both DRB1*07 and DQB1*02 were significant risk alleles in the association analysis, we performed a conditional haplotype analysis on HLA‐DRB1*07‐DQB1*02 to check whether they were independent risk factors. The association with HLA‐DRB1*07 was no longer significant after controlling for DQB1*02, whereas DQB1*02 without the DRB1*07 background showed a weak negative association. The latter finding can be explained by DQB1*02 linkage with the “protective” alleles DRB1*03 and DRB1*11. Overall, these results suggest a major role for the HLA‐DRB1*07‐ DQB1*02 haplotype, making it the main genetic predisposing factor to LOMG in our population. Consistently, the LD between DRB1*07 and DQB1*02 was higher among patients than controls (D' = 0.771 vs D' = 0.341).

We also found that the DRB1*07 frequency increased in parallel with age at onset: it was higher among patients aged 80 years or older (28.6%) than in those aged 60‐79 years (25.4%) or 50‐59 years (14.6%). The average age at onset was 73.4 years among homozygotes for DRB1*07, 69.7 among heterozygotes and 66.6 among patients not carrying any DRB1*07 allele (*P* = 0.0488) (Fig S1). The haplotype DRB1*07‐DQB1*02 showed an association trend with age of onset (*P* = 0.0626).

To investigate whether DRB1*07 may serve as a disease biomarker in LOMG, we compared other demographic characteristics and clinical aspects, between carriers (n = 45) and non‐carriers (n = 62). All the analyzed variables did not differ between the two groups (Table S2).

Titin Abs were assayed in serum samples from 70 patients and were detected in 61.4% (43/70). No association was found between titin Ab positivity and age at onset or any other demographic or clinical features (Table S3). HLA‐DRB1*07 was more frequent among titin‐positive than ‐negative patients (11/43 vs 4/27, *P* = 0.37). The association of titin Abs with the haplotype DRB1*07‐DQB1*02 reached borderline significance (*P* = 0.0504). No associations of titin Abs with any other HLA allele or haplotype were found.

## DISCUSSION

MG is a heterogeneous disease when considering associated Abs, thymus pathology and age of onset.^2^ As in other autoimmune diseases, HLA Class I and II antigens are the main predisposing factors. EOMG is strongly linked with the extended haplotype 8.1 (A1‐B8‐DQ2‐DR3) in Caucasians,^10^, ^11^, ^12^, ^13^ whereas MuSK‐MG is associated with DQB1*05 and HLA‐DRB1*14/DRB1*16 across different populations.^28^, ^29^, ^30^, ^31^ In LOMG, results have been far less consistent: initially, an association with HLA‐B7 and DR2 was reported.^10^, ^11^ More recently, this disease subtype was found to be strongly associated with DRB1*15:01 in a large sample of Norwegian MG patients ≥60 years at disease onset.^12^ Other studies, conducted in cohorts of different ethnicities and often of small size, reported mixed results: an association with DRB1*04 was found in Tunisians,^14^ with DRB1*01 in Portuguese,^15^ with DQA1*02:01, DQA1*01:02, and DQB1*06:02 in Turkish^16^ and with DQB1*05:02 and DRB1*16 in Italian MG patients.^18^, ^19^ In two GWAS, LOMG was associated with different signals. In the former, conducted in U.S. patients, there was an association with *TNFRSF11A* (rs4263037) and with rs9271871 in the DRB1‐DQA1 intergenic region.^21^ The latter, performed in North European patients, found an association with DRB1*07:01, DRB1*15:01, and DQB1*02:02, which, however, did not reach genome‐wide significance, whereas DRB1*03:01, DRB1*13:01, and DQB1*02:01 were found to be protective alleles.^20^

In this study, we investigated HLA Class II associations in a large cohort of patients with AChR Ab positive, generalized, non‐thymoma LOMG of single ethnicity. We found that HLA‐DRB1*07 and, to a lesser extent, DRB1*14 increased the disease risk. DRB1*07 was previously associated with LOMG in Northern European patients^20^, ^32^: therefore, our results extend this association to South Europe. Regarding the *DQB1* locus, Seldin and coworkers found that HLA DQB1*02 had opposite effects: DQB1*02:01 was protective, whereas DQB1*02:02 was a risk factor.^20^ The DQB1*02 was associated with LOMG in our study, that, however, was not powered to point out differences at the 4‐digit level (data not shown). Overall, these results support the role of HLA DRB1*07 and DQB1*02 as risk alleles for LOMG across Caucasian populations.

Interestingly, we found a novel negative association with HLA‐DRB1*11, suggesting a potential protective role of this allele, as already documented in other autoimmune diseases.^33^, ^34^ Our data also confirmed the negative association of LOMG with HLA‐DRB1*03, as reported in previous studies.^12^, ^20^ This allele is part of the ancestral haplotype 8.1 (HLA A1‐B8‐D3‐DQ2) and is strongly associated with EOMG, as well as other autoimmune diseases.^35^ The finding that EOMG and LOMG show opposite associations with DRB1*03, suggests multiple mechanisms leading to tolerance breakdown in these MG subtypes. These may include an interference in the determinants capture during self‐antigen presentation,^36^ a modification of the selected self‐epitopes by HLA alleles/haplotypes combinations,^37^ or a modulation of suppressor T regulatory cells.^38^

In our cohort, the DRB1*07‐DQB1*02 haplotype showed the strongest association with the disease. Indeed, the conditional haplotype analysis revealed that neither DRB1*07 nor DQB1*02 independently predisposed to LOMG, whereas the entire haplotype conferred an increased risk. As a result of the increased frequency of these two alleles in our cohort, the LD between them was higher in patients than in controls (D' = 0.771 vs D' = 0.341). Based on these findings, it may be hypothesized that neither DRB1*07 nor DQB1*02 are the causative alleles. Rather, one or more other loci in linkage with them on the same identified haplotype, such as HLA *DQA1,* a Class III gene or a gene located outside the HLA region, might be the actual predisposing factor to LOMG. Further studies are needed to clarify this point.

In addition, we found an association with DRB1*14‐DQB1*05 and DRB1*15‐DQB1*06 haplotypes. However, due to their weaker statistical association (p = 0.0012 and p = 0.0018, respectively) and their low frequency in the control population (0.0225 and 0.018 respectively), they were considered less likely to be predisposing factors.

Recently, an increased frequency of DRB1*16 and DQB1*05 was reported in an Italian cohort of 49 LOMG patients.^18^, ^19^ We could not replicate these findings for DQB1*05 nor for DRB1*16 as single alleles, however we detected a weak association for the haplotype DRB1*16‐DQB1*05 (0.039 in controls vs 0.07 in LOMG, *P* = 0.0494). Supposing that these two patient cohorts have the same genetic background, differences in inclusion criteria and age at onset cut‐off may explain these divergent results.

Renton and co‐workers found that the association of the *TNFRSF11A* single nucleotide polymorphism with MG increased in parallel with age of onset.^21^ Similarly, in our population, the DRB1*07 carrier frequency increased with the age at onset, and the median onset age was higher for homozygotes compared to heterozygotes and DRB1*07 non‐carriers.

Giraud et al. reported the association of HLA‐DR7 with titin Abs in non‐thymoma French MG patients.^32^ As titin Abs are frequently detected in non‐thymoma LOMG, this finding indirectly supports the association between DR7 and this MG subtype, possibly not detected in their study because of the low cut‐off age (40 years). In line with this view, it was suggested that titin Abs and DR7 may better identify the LOMG subtype.^39^, ^40^ However, in our study we did not find a significant association between HLA‐DRB1*07 and titin Abs, rather an association trend for the haplotype DRB1*07‐DQB1*02. The small number of patients tested for titin Abs likely underpowered our analysis. When clinical characteristics were analyzed, DRB1*07 carriers and non‐carriers did not differ in disease severity, comorbidities, and response to therapies by univariate analysis. Furthermore, in our cohort, titin Abs were not associated with a more severe disease course, in line with other studies performed in non‐Scandinavian populations,^41^ questioning their utility as a negative prognostic marker. These Abs are commonly detected in MG‐thymoma, their production being most likely triggered by the tumour.^42^ On the other hand, the mechanisms leading to titin and RyR Abs production in LOMG are yet to be clarified.

One of the strongest points of our study was the inclusion of only non‐thymoma, late‐onset, generalized MG patients positive for AChR Abs in order to analyze a more homogenous population and possibly overcome the inherent heterogeneity of LOMG. The consecutive enrollment conceivably reduced the risk of selection bias. Moreover, the large size of our cohort and the use of publicly available controls, representative of the entire Italian population, reduced the risk of sampling error. On the other hand, a major study limitation is that we analyzed only *DRB1* and *DQB1* loci, hence it is possible that other loci, or genes, are responsible for or contribute to LOMG susceptibility. Unfortunately, neither HLA typing, nor titin Ab testing, provided valuable markers for disease prognosis and management. However, our data support the role of the haplotype HLA DRB1*07‐DQB1*02 as a genetic risk factor for generalized AChR‐positive non‐thymoma LOMG in the Italian population. Further studies, based on a more comprehensive genomic analysis, are required to further clarify the predisposing factors in LOMG and their role in disease pathogenesis and management.

## Funding Information

This study was funded by the “Linea D.1” grant of the Catholic University of Sacred Heart (Rome, Italy), grant code R4124500986, and by the Italian Ministry of Health Grant No. RF‐2016‐02364384.

## Authors’ Contribution

GS: drafting of the manuscript; acquisition, analysis, and interpretation of data; critical revision of the manuscript for important intellectual content. LT, GM, MV, GDS, VD, MM: acquisition, analysis, and interpretation of data; critical revision of the manuscript for important intellectual content. AE: study concept and design; acquisition, analysis, and interpretation of data; critical revision of the manuscript for important intellectual content. FL: analysis and interpretation of data; critical revision of the manuscript for important intellectual content. CP: drafting of the manuscript; acquisition, analysis, and interpretation of data; critical revision of the manuscript for important intellectual content.

## Conflict of Interest

The authors report no disclosures.

## Supporting information


**Table S1**. Comparative analysis between the included cohort and total LOMG population treated at our Center.
**Table S2**. Comparative analysis of demographic and clinical features of DRB1*07 carriers and non‐carriers.
**Table S3**. Comparative analysis of demographic and clinical features of titin Ab positive and negative patients.
**Table S4**. Haplotype frequencies in LOMG patients and controls.
**Figure S1**. Mean age at disease onset in patients carrying or not carrying the HLA‐DRB1*07 allele. The average age at onset was 73.4 years among homozygotes for DRB1*07 (DRB1*07 H), 69.7 among heterozygotes (DRB1*07 HT), and 66.6 among patients not carrying any DRB1*07 allele (NO DRB1*07) (p = 0.0488). H = homozygote; HT = heterozygote; bars = standard mean errors.
