# Supplementary material for: Human Leukocyte Antigen Class II associations in late‐onset Myasthenia Gravis
Source: Ann Clin Transl Neurol. 2021 Feb 5;8(3):656–65. doi: 10.1002/acn3.51309 (PMC7951107; doi:10.1002/acn3.51309)
Supplement: Supplementary file 1 — Table S1. Comparative analysis between the included cohort and total LOMG population treated at our Center. Table S2. Comparative analysis of demographic and clinical features of DRB1*07 carriers and non‐carriers. Table S3. Comparative analysis of demographic and clinical features of titin Ab positive and negative patients. Table S4. Haplotype frequencies in LOMG patients and controls. Figure S1. Mean age at disease onset in patients carrying or not carrying the HLA‐DRB1*07 allele. The average age at onset was 73.4 years among homozygotes for DRB1*07 (DRB1*07 H), 69.7 among heterozygotes (DRB1*07 HT), and 66.6 among patients not carrying any DRB1*07 allele (NO DRB1*07) (p = 0.0488). H = homozygote; HT = heterozygote; bars = standard mean errors. [file ACN3-8-656-s001.docx]

|  | **Included Patients  N=107** | **Total LOMG cohort**  **N=254** | **p** |
| --- | --- | --- | --- |
| Median age at onset, years (range) | 68 (50-92) | 68 (50-92) | NS |
| Females | 28 (26.2%) | 76 (29.9%) | NS |
| **Maximum MGFA** | | | |
| II-III | 77 (72%) | 206 (81.1%) | NS |
| IV-V | 30 (28%) | 48 (18.9%) | NS |
| **Geographical origin** | | | |
| North | 8 (7.5%) | 16 (6.3%) | NS |
| Centre | 57 (53.3%) | 127 (50%) | NS |
| South | 39 (36.4%) | 105 (41.3%) | NS |
| Sardinia | 3 (2.8%) | 6 (2.4%) | NS |

**Table S1. Comparative analysis between the included cohort and total LOMG population treated at our Centre.**

*MGFA: Myasthenia Gravis Foundation of America classification.*

**Table S2. Comparative analysis of demographic and clinical features of DRB1*07 carriers and non-carriers.**

|  | **DRB1*07 carriers**  **N=45** | **DRB1*07 non-carriers**  **N=62** | **p** |
| --- | --- | --- | --- |
| **Females** | 9 (20%) | 19 (30.6%) | NS |
| **Max MGFA** | | | |
| **II** | 18 (40%) | 20 (32.3%) | NS |
| **III** | 15 (33.3%) | 24 (38.7%) | NS |
| **IV** | 7 (15.6%) | 8 (12.9%) | NS |
| **MG Crisis** | 5 (11.1%) | 10 (16.1%) | NS |
| **Bulbar phenotype** | 31 (68.9%) | 44 (71%) | NS |
| **Post-intervention Status** | | | |
| D, W, U | 1 (2.2%) | 2 (3.2%) | NS |
| I | 15 (33.3%) | 13 (21%) | NS |
| MM or PR | 27 (60%) | 45 (72.6%) | NS |
| CSR | 2 (4.4%) | 2 (3.2%) | NS |
| **Autoimmune comorbidities** | 7 (15.6%) | 6 (9.7%) | NS |
| **Therapy used** | | | |
| Prednisone | 10 (22.2%) | 12 (19.4%) | NS |
| Prednisone +IS | 30 (66.7%) | 44 (71%) | NS |
| Pyridostigmine alone | 5 (11.1%) | 6 (9.7%) | NS |

*MGFA: Myasthenia Gravis Foundation of America classification; D: Died of MG; W: worsened; U: unchanged; I: improved; MM: minimal manifestations; PR: pharmacological remission; CSR: complete stable remission; IS: immunosuppressant drug.*

|  | **Titin Ab +**  **N=43** | **Titin Ab –**  **N=27** | **p** |
| --- | --- | --- | --- |
| **Age at onset, median,** years (range) | 66 (50-92) | 69 (50-86) | NS |
| **Females** | 8 (18.6%) | 10 (37%) | NS |
| **DRB1*07+** | 11 (25.6%) | 4 (14.8%) | NS |
| **Max MGFA** | | | |
| II | 16 (37.2%) | 8 (29.6%) | NS |
| III | 19 (44.2%) | 11 (40.7%) | NS |
| IV | 6 (14%) | 3 (11.1%) | NS |
| **MG Crisis** | 2 (4.7%) | 5 (18.5%) | NS |
| **Bulbar phenotype** | 29 (67.4%) | 19 (70.4%) | NS |
| **Post-intervention Status** | | | |
| D, W, U | 2 (4.7%) | 0 | NS |
| I | 8 (18.6%) | 8 (29.6%) | NS |
| MM or PR | 31 (72.1%) | 19 (70.4%) | NS |
| CSR | 2 (4.7%) | 0 | NS |
| **Autoimmune comorbidities** | 8 (18.6%) | 1 (3.7%) | NS |
| **Therapy used** | | | |
| prednisone alone | 10 (23.3%) | 6 (22.2%) | NS |
| prednisone + IS | 27 (62.8%) | 17 (63%) | NS |
| Pyridostigmine alone | 6 (14%) | 4 (14.8%) | NS |

**Table S3. Comparative analysis of demographic and clinical features of titin Ab positive and negative patients.**

*Ab: antibody; MGFA: Myasthenia Gravis Foundation of America classification; D: Died of MG; W: worsened; U: unchanged; I: improved; MM: minimal manifestations; PR: pharmacologic remission; CSR: complete stable remission; IS: immunosuppressant drug.*

**Table S4. Haplotype frequencies in LOMG patients and controls.**

|  | **Controls N=1411** | | **LOMG, N=107** | | | | | **p values** | |  |
| --- | --- | --- | --- | --- | --- | --- | --- | --- | --- | --- |
|  | **Alleles (#)** | **Freqency** | **Alleles**  **(#)** | **Freqency** | **WT** | **HT** | **H** | **Fisher’s Exact Test** | **FDR** | **OR (95% C.I.)** |
| DRB1*01-DQB1*05 | 181 | 0.063 | 17 | 0.079 | 91 | 15 | 1 | 0.3876 | 0.9342 | 1.26 (0.75-2.11) |
| DRB1*03-DQB1*02 | 153 | 0.053 | 5 | 0.023 | 102 | 5 | 0 | 0.0538 | 0.3647 | 0.42 (0.17-1.03) |
| DRB1*04-DQB1*02 | 23 | 0.008 | 2 | 0.010 | 105 | 2 | 0 | 0.6948 | 1.0000 | 1.15 (0.27-0.49) |
| DRB1*04-DQB1*03 | 151 | 0.052 | 16 | 0.074 | 91 | 16 | 0 | 0.2107 | 0.6766 | 1.43 (0.84-2.44) |
| DRB1*07-DQB1*02 | 150 | 0.052 | 40 | 0.184 | 70 | 34 | 3 | **6.01x10^-11^** | **3.67x10^-9^** | **4.10 (2.80-5.99)** |
| DRB1*07-DQB1*03 | 106 | 0.037 | 10 | 0.050 | 98 | 8 | 1 | 0.4598 | 1.0000 | 1.26 (0.65-2.44) |
| DRB1*08-DQB1*03 | 26 | 0.009 | 2 | 0.007 | 105 | 2 | 0 | 1.0000 | 1.0000 | 1.01 (0.24-4.30) |
| DRB1*08-DQB1*04 | 43 | 0.015 | 4 | 0.019 | 103 | 4 | 0 | 0.5703 | 1.0000 | 1.23 (0.44-3.46) |
| DRB1*10-DQB1*05 | 35 | 0.012 | 3 | 0.014 | 104 | 3 | 0 | 0.7477 | 1.0000 | 1.13 (0.35-3.71) |
| DRB1*11-DQB1*03 | 537 | 0.186 | 35 | 0.163 | 77 | 25 | 5 | 0.3653 | 0.9342 | 0.83 (0.57-1.21) |
| DRB1*11-DQB1*05 | 79 | 0.027 | 1 | 0.005 | 106 | 1 | 0 | **0.0422** | 0.3647 | 0.16 (0.02-1.18) |
| DRB1*12-DQB1*03 | 28 | 0.010 | 2 | 0.009 | 105 | 2 | 0 | 1.0000 | 1.0000 | 0.94 (0.22-3.98) |
| DRB1*13-DQB1*03 | 89 | 0.031 | 3 | 0.014 | 104 | 3 | 0 | 0.2107 | 0.6766 | 0.44 (0.14-1.39) |
| DRB1*13-DQB1*06 | 172 | 0.060 | 21 | 0.097 | 87 | 19 | 1 | **0.0406** | 0.3647 | 1.68 (1.04-2.70) |
| DRB1*14-DQB1*02 | 21 | 0.007 | 1 | 0.005 | 106 | 1 | 0 | 1.0000 | 1.0000 | 0.63 (0.08-4.68) |
| DRB1*14-DQB1*05 | 108 | 0.037 | 19 | 0.089 | 88 | 19 | 0 | **0.0012** | **0.0364** | **2.45 (1.47-4.07)** |
| DRB1*15-DQB1*05 | 29 | 0.010 | 2 | 0.008 | 105 | 2 | 0 | 1.0000 | 1.0000 | 0.91 (0.22-3.83) |
| DRB1*15-DQB1*06 | 82 | 0.028 | 16 | 0.076 | 91 | 16 | 0 | **0.0018** | **0.0364** | **2.70 (1.55-4.70)** |
| DRB1*16-DQB1*05 | 113 | 0.039 | 15 | 0.070 | 94 | 11 | 2 | **0.0494** | 0.3647 | 1.81 (1.03-3.16) |
| other | 696 | 0.240 |  |  |  |  |  |  |  |  |
| total | 2822 | 0.975 | 214 | 1.000 |  |  |  |  |  |  |
| missing | 72 | 0.025 | 0 | 0,000 |  |  |  |  |  |  |

*Controls are from Rendine et al., ref. 25; LOMG= late-onset myasthenia gravis; WT=wild type; H= homozygote; HT= heterozygote; #= number of alleles; FDR: Fisher's exact test with FDR correction; OR: odds ratio; C.I.= confidence interval; other: all haplotypes that were not present in LOMG cohort.*


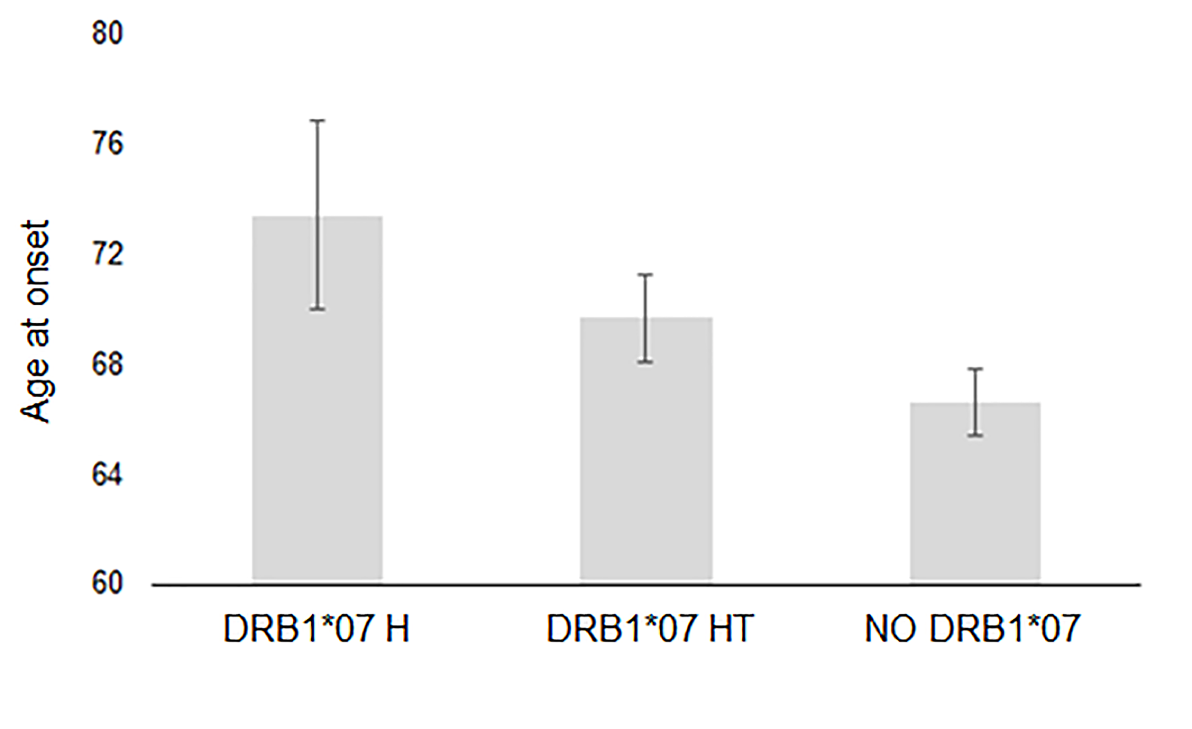


**Figure S1. Mean age at disease onset in patients carrying or not carrying the HLA-DRB1*07 allele.** The average age at onset was 73.4 years among homozygotes for DRB1*07 (DRB1*07 H), 69.7 among heterozygotes (DRB1*07 HT) and 66.6 among patients not carrying any DRB1*07 allele (NO DRB1*07) (p=0.0488). H= homozygote; HT= heterozygote; bars = standard mean errors.
